# Innate Immune Response After Cardiac Arrest (INNATUS): A Study Protocol for an Observational Single‐Center Pilot Study

**DOI:** 10.1111/aas.70087

**Published:** 2025-07-08

**Authors:** Asser M. J. Seppä, Huai Hui Wong, Luz E. Cabrera, Dawit A. Yohannes, Maria Heliste, Marjaana Tiainen, Johanna Hästbacka, Markus B. Skrifvars, Eliisa Kekäläinen, Pirkka T. Pekkarinen

**Affiliations:** ^1^ Division of Intensive Care Medicine, Department of Anaesthesiology, Intensive Care and Pain Medicine University of Helsinki and Helsinki University Hospital Helsinki Finland; ^2^ Department of Bacteriology and Immunology University of Helsinki Helsinki Finland; ^3^ Translational Immunology Research Program Faculty of Medicine, University of Helsinki Helsinki Finland; ^4^ Perioperative and Intensive Care University of Helsinki and Helsinki University Hospital Helsinki Finland; ^5^ Department of Neurology Helsinki University Hospital and University of Helsinki Helsinki Finland; ^6^ Department of Intensive Care Tampere University Hospital, Wellbeing Services County of Pirkanmaa Tampere Finland

**Keywords:** cardiac arrest, inflammation, innate immunity, resuscitation, systems immunology

## Abstract

**Background:**

The treatment of cardiac arrest (CA) patients is often complicated by post‐cardiac arrest syndrome (PCAS), which involves a systemic inflammatory response. Increased levels of certain inflammatory markers (e.g., interleukin‐6 and procalcitonin) are associated with higher mortality and worse neurological outcomes. Previous studies have focused on the prognostic value of individual markers, whereas the interplay between inflammatory mediators in the setting of CA remains largely unclear. In this study, we aim to examine the patterns of inflammatory markers and their association with the severity of organ failure and 6‐month neurological outcomes in adult CA patients.

**Methods:**

This is a prospective observational single‐center study. The study cohort consists of 40 adult CA patients admitted to the ICU after successful resuscitation, and a control group of 40 patients undergoing elective coronary artery bypass graft (CABG) surgery. Patient enrollment started in January 2022 and was completed in April 2025. The last follow‐up samples and interviews are due in October 2025. We collect blood samples upon ICU admission, at 4, 8, 12, and 24 h after return of spontaneous circulation (ROSC) and on the mornings of treatment Days 1–4. We combine clinical data with proteomic, transcriptomic, and flow cytometric analyses of blood samples to generate a comprehensive systems immunology landscape of the PCAS. The control group of CABG patients is used to assess inflammatory changes related to surgical trauma and ICU treatment in general, with the goal of distinguishing inflammatory disturbances specific to CA. This allows the identification of complex inflammatory pathways relevant to the pathogenesis of PCAS. The gathered information can then be used to plan future anti‐inflammatory interventional trials. The primary outcomes of the study are the severity of organ failure during the first 96 h after ROSC and neurological outcome at 6 months. We will assess these using a modified SOFA score and the Modified Rankin Scale, respectively. We will use principal component analysis, latent profile analysis, and hierarchical clustering to identify patient subgroups with similar immunological response profiles. We will assess the correlation of different immunological response profiles with primary outcomes using linear mixed‐effects models.

## 1 Introduction

Treatment of patients successfully resuscitated from cardiac arrest (CA) is often complicated by post‐cardiac arrest syndrome (PCAS), which comprises myocardial dysfunction, hypoxic–ischemic brain injury, ischemia–reperfusion injury, and the persistence of the pathological process that caused CA [1]. Systemic inflammation in response to the whole‐body ischemia–reperfusion injury is involved in its pathogenesis [1]. Currently, no high‐quality evidence supports a specific treatment that improves PCAS outcomes beyond standard intensive care unit (ICU) management [2]. Prognostication and decisions regarding withdrawal of care rely on a multimodal approach, including clinical examination, electroencephalography, somatosensory evoked potentials, neuron‐specific enolase, and neuroimaging [2].

The inflammatory response involved in PCAS is characterized by elevated circulating levels of numerous inflammatory biomarkers, such as procalcitonin and interleukin‐6 (IL‐6) [3, 4, 5]. The origin and triggers of this inflammatory response remain poorly understood. Although several studies have investigated the presence and prognostic value of individual inflammatory markers after CA, the complex interplay among different immunologic mediators has received less attention. Anti‐inflammatory interventions have been explored in some clinical trials of post‐arrest care, but thus far, they have not resulted in clinically significant improvements in mortality or neurological outcome [6, 7]. For example, early administration of methylprednisolone after return of spontaneous circulation (ROSC) has been associated with lower IL‐6 levels [7], while the administration of tocilizumab, an IL‐6 receptor antagonist, has been shown to lower C‐reactive protein (CRP) and leukocyte counts [6]. These findings suggest that post‐CA immune activation is a modifiable factor, but identifying optimal targets for future therapeutic strategies requires a more comprehensive understanding of the complex inflammatory response after CA.

Innate immune cells are among the earliest responders to ischemia–reperfusion injury, even in the absence of microbial pathogens [8]. In addition to innate‐like lymphoid cells, which can rapidly activate and amplify immune responses independently of antigen presentation, myeloid cells play a central role in shaping the early systemic immune landscape [9, 10]. Among these, monocytes and neutrophils, including low density granulocytes (LDGs), have gained particular attention. Their subset distribution, activation status, and population dynamics may reflect underlying pathophysiological processes and carry relevance for disease severity and progression. In this study, we investigate both lymphoid and myeloid components of the evolving immune response, with the purpose of characterizing their early post‐resuscitation alterations, as well as exploring their link with clinically meaningful parameters. We specifically focus on circulating monocytes, neutrophils, LDGs, and innate lymphoid populations (natural killer [NK] cells, mucosal‐associated invariant T [MAIT] cells, and γδ‐T cells), as well as markers of systemic redox balance (nicotinamide adenine dinucleotide [NAD] and glutathione [GSH] metabolites). Additionally, we aim to elucidate the temporal dynamics and the interplay between inflammatory mediators to better understand immunologic patterns, rather than examining associations between individual markers and outcomes.

## 2 Methods

### 2.1 Design and Setting

The INNATUS study is a prospective, observational single‐center pilot study of adult patients resuscitated from CA. The study is conducted across three ICUs at a single university hospital campus (Meilahti Hospital, Helsinki University Hospital, Helsinki, Finland). In this cohort, we assess immune responses through the analysis of cell surface and intracellular markers, as well as proteomic and transcriptomic profiling. The primary outcomes are the severity of organ failure during the first 96 h of post‐resuscitation care and neurological outcome at 6 months following CA. Patient recruitment began in January 2022 and was completed in April 2025. The last follow‐up samples and interviews are due in October 2025. The study protocol has been approved by the Helsinki University Hospital Ethics Committee (HUS/3028/2020, November 18th, 2020) and is conducted in accordance with the Declaration of Helsinki. We will report the results according to the Strengthening the Reporting of Observational Studies in Epidemiology (STROBE) guidelines [11].

### 2.2 Study Population

The study population consists of 40 successfully resuscitated adult patients (age ≥ 18 years) treated in the participating ICUs within Helsinki University Hospital following CA, and a control group of 40 patients undergoing elective coronary artery bypass graft (CABG) surgery. Samples collected from the CABG control group after induction of anesthesia but before surgery will be used to assess baseline levels of immunological variables. Samples collected from the CABG control group during the postoperative treatment period in the ICU will be compared to those from the CA group. The rationale is to distinguish inflammation related to surgical trauma, local ischemia–reperfusion injury of the heart, epinephrine (adrenaline) administration, and general ICU treatment in the CABG group from the specific features of inflammatory activation related to whole‐body ischemia–reperfusion injury, tissue damage, and injury caused by cardiopulmonary resuscitation (e.g., chest trauma due to compressions and pulmonary injury due to high‐pressure ventilation with 100% oxygen). In the CABG group, the moment of weaning from intraoperative cardiopulmonary bypass will be used as the reference time point comparable to ROSC in the CA group to align sampling at 4, 8, 12, and 24 h after ROSC.

### 2.3 Inclusion and Exclusion Criteria

The inclusion criteria for the study participants are as follows: [1] age ≥ 18 years and [2] admission to a participating ICU at Helsinki University Hospital after successful resuscitation with ROSC or scheduled CABG surgery at Helsinki University Hospital with planned postoperative admission to a participating ICU. The exclusion criteria are as follows: [1] age < 18 years; [2] pregnancy or lactation at the time of enrollment; [3] admission of a CA patient to a participating ICU more than 2 h after ROSC; [4] status as a prisoner or forensic psychiatry patient; [5] inability to consider informed consent due to a mental health disorder or intellectual disability; [6] consent not likely to be obtained (e.g., no available legal representative); and [7] known prior refusal to participate in clinical studies or refusal to participate in this study.

### 2.4 Consent to Participate

CA patients will be enrolled in the study immediately upon admission to the ICU, and deferred informed consent will be sought from the next of kin as soon as possible. If the patient recovers to sufficient cognitive status, informed consent will be sought from the patient during the hospital stay. For CABG patients, informed consent will be obtained during the preoperative visit to the hospital.

### 2.5 Sample Collection and Handling

Blood samples for proteomics, transcriptomics, and flow cytometry will be collected from the arterial line at the following time points: upon admission, at 4, 8, 12, and 24 h after ROSC, as well as on the mornings of Days 1, 2, 3, and 4 (D1–4) of post‐resuscitation ICU care. Isolation of peripheral blood mononuclear cells (PBMCs) and central venous blood sampling will be carried out twice during the ICU stay on working days (e.g., weekdays), preferably on the mornings of D1 and D3 (Table 1). Recovery samples will be collected from a peripheral vein at 6–12 months after CA or CABG surgery.

**TABLE 1:** Sample collection time points.

		Purpose	Before surgery (CABG group only)	ICU admission	4 h after ROSC	8 h after ROSC	12 h after ROSC	24 h after ROSC	Morning on Days 1–4
Clinical samples	CBC with differential	Leukocyte main classes	x	x					x
	ABG		x	x	x	x	x	x	x
	cVBG		x						x*
	Heparin‐plasma	LD, Ferritin	x	x					x
Study samples (arterial line)	3 mL serum gel tube	Protein markers (e.g., cytokines)	x	x	x	x	x	x	x
	2.5 mL RNA preservation (PAXgene) tube	RNA seq	x	x	x	x	x	x	x
	Cryopreserved (PROT‐1) Heparin WB	Flow cytometry	x	x	x	x	x	x	x
	6 mL EDTA	Protein markers (e.g., complement)	x	x					x
	2 mL EDTA	Redox	x	x					x*
	3 × 9 mL heparin tube	PBMC isolation, flow cytometry, etc.	x						x*
Study samples (central venous line)	6 mL serum tube	Protein markers (e.g., cytokines)	x						x*
	Cryopreserved (PROT‐1) Heparin WB	Flow cytometry	x						x*
	4 mL EDTA	Protein markers (e.g., complement)	x						x*
	3 × 9 mL heparin tube	PBMC isolation, flow cytometry, etc.	x						x*

*Note:* Pre‐surgery samples are collected after induction of anesthesia and insertion of an arterial cannula and central venous catheter. At this time point, PBMC, plasma and serum samples are taken both from the arterial cannula and the central venous catheter. In the CABG group, the moment of weaning from intraoperative cardiopulmonary bypass is considered analogous to ROSC. x, sampling time point. x*, larger sampling performed twice on working day mornings during D1–D4, preferably D1 and D3. Basic sampling is performed from arterial cannula. On the occasions of larger sampling, PBMC, plasma, and serum samples are drawn both from the arterial cannula and central venous catheter. D1–D4 samples are taken as long as the patient is still in the ICU.

Abbreviations: ABG, arterial blood gas; CABG, coronary artery bypass graft; CBC, complete blood count; cVBG, central venous blood gas; EDTA, ethylenediaminetetraacetic acid; LD, lactate dehydrogenase; PBMC, peripheral blood mononuclear cells; RNA, ribonucleic acid; ROSC, return of spontaneous circulation; WB, whole blood.

## 3 Immunological Analysis

This study will apply a multi‐omics systems immunology approach in a clinically well characterized patient cohort. We will use multiplexed protein measurements to measure soluble inflammatory markers, such as cytokines and complement factors. We will measure the oxidized and reduced forms of nicotinamide adenine dinucleotide NAD and GSH in blood to assess systemic redox balance. We will use multiple flow cytometry panels to study the cellular part of immunity. Funding allowing, we will perform a whole blood transcriptome analysis to identify cellular activation pathways overly activated by PCAS and broaden the proteomic analysis with liquid chromatography–mass spectrometry‐based analysis of enriched plasma proteins, allowing the simultaneous analysis of up to 5000 different proteins.

## 4 Clinical and Baseline Data Collection

The following baseline data will be collected from all enrolled patients: age, sex, height, weight, current smoking status, New York Heart Association functional class prior to CA, level of independence in self‐care prior to CA, use of systemic immunosuppressive medication, use of inhaled corticosteroids, and presence of known infection prior to CA. Resuscitation characteristics will be recorded according to Utstein criteria, including the site of arrest, presence of bystander resuscitation, initial rhythm, ROSC delay, cumulative epinephrine dose administered, known or suspected aspiration of gastric content prior to airway intubation, and coronary angiography and/or percutaneous coronary intervention within 24 h from ROSC.

Data collected during the post‐resuscitation care and treatment will include daily clinical laboratory values, vasopressor and inotrope doses, intubation status, time of intubation and extubation, FiO_2_, SpO_2_, body temperature, use of therapeutic hypothermia (TH), TH start and end times, possible antimicrobial treatment start and end times, indication of antimicrobial therapy, hospital discharge vital status (or on Day 30 if still hospitalized), and any limitations of treatment during the hospital stay (up to 30 days).

## 5 Outcomes

The modified SOFA (mSOFA) score used in this study is based on the original SOFA scoring system published by Vincent et al. [12], with a modification to the cardiovascular subscore, which is extended to a 6‐point scale (Table 2). The rationale behind this adaptation is to more accurately reflect the increased use of vasopressors in modern post‐resuscitation care, which has diluted the ability of the original SOFA score to detect cardiovascular failure [13] To compensate for this, we have extended the cardiovascular subscoring to account for this increased need for vasopressor support. A similar approach was previously applied by Annborn et al. [14].

**TABLE 2:** Modified SOFA score.

mSOFA subscore	1	2	3	4	5	6
Respiration, PaO_2_/FiO_2_ (mmHg)	< 400	< 300	< 200 (and mechanical ventilation)	< 100 (and mechanical ventilation)	N/A	N/A
Coagulation, platelets × 10^3^/mm^3^	< 150	< 100	< 50	< 20	N/A	N/A
Hepatic, bilirubin (mg/dL)	1.2–1.9	2.0–5.9	6.0–11.9	> 12.0	N/A	N/A
Cardiovascular (vasoactive agents μg/kg/min, for at least 1 h)	MAP < 70 mmHg	Dopamine ≤ 5 or dobutamine (any)	Dopamine > 5 norepinephrine or epinephrine ≤ 0.1	Dopamine > 15 norepinephrine or epinephrine > 0.1 but ≤ 0.2^a^	Norepinephrine or epinephrine > 0.2 but ≤ 0.3^a^	Norepinephrine or epinephrine > 0.3^a^
Neurological, glasgow coma scale	13–14	10–12	6–9	< 6	N/A	N/A
Renal, creatinine (mg/dL) or urine output (mL/day)	1.2–1.9	2.0–3.4	3.5–4.9 or < 500	> 5.0 or < 200	N/A	N/A

*Note:* Compared to the original SOFA score there are extensions in the cardiovascular subscoring for patients with most severe hemodynamic instability.

^a^ Modified compared to the original SOFA scoring.

The mSOFA score will be assessed upon ICU admission based on the most severe component values recorded from admission to ROSC +4 h, and on the mornings of D1–4 in the ICU as the most severe values recorded during the preceding 24 h. If there are missing values for some mSOFA components during the ICU stay, the previous day's value will be carried forward to calculate mSOFA. If a discharge to a hospital ward occurs before D4, mSOFA is recorded until D4, but missing values during ward stay will be assumed normal. In the event of death or a decision to withdraw all life‐sustaining treatment, the last recorded mSOFA score plus 2 points will be used for subsequent time points until D4.

The primary outcomes of the study are: (1) mSOFA and its change as a function of time recorded at the beginning of ICU treatment and daily during the first 96 h after ROSC; and (2) Modified Rankin Scale (mRS) at 6 months after CA, assessed via structured telephone interview by a certified neurologist.

The secondary outcomes are: (1) highest value of mSOFA during the entire ICU or other critical care stay, until discharge from the ICU; (2) composite outcome of in‐hospital mortality or any new treatment‐limitation decision during the hospital admission up to 30 days; (3) in‐hospital mortality within 30 days; (4) any new decision to limit or withdraw treatment during hospital admission up to 30 days; (5) length of ICU stay; (6) length of hospital stay; (7) suspected or confirmed secondary infection requiring initiation or escalation of antimicrobial treatment > 72 h after ROSC during hospitalization (up to 30 days); and (8) Cerebral Performance Category (CPC) at 6 months after CA, assessed via structured telephone interview by a certified neurologist.

Poor neurological outcome is defined as mRS 4–6 or CPC 3–5. However, we will use the continuous scale of neurological outcome without dichotomization in statistical analyses whenever possible.

## 6 Statistical Analysis

We will start the statistical analysis of the primary publication by exploring the data collected during the first 24 h after ROSC. For CABG patients, the end of perfusion during surgery is used as the reference time point, analogous to ROSC in CA patients. In addition, CABG patients will have a preoperative sampling time point after induction of anesthesia but before surgery that will serve as a baseline for immunological variables in this cohort.

During this exploration phase, we will identify and address issues such as a high frequency of values below the assay detection limit. We will also assess the distribution and scale of each variable to ensure comparability. Where appropriate, methods such as standardization (*z*‐scores based on mean and standard deviation) and logarithmic transformation will be applied. After addressing data quality and comparability, we will identify variables that correlate with the primary outcomes (mSOFA and 6‐month mRS). Our hypothesis is that poorly regulated inflammation contributes to acute organ dysfunction, which in turn causes secondary neurological damage, ultimately affecting long‐term neurological recovery, as measured by the 6‐month mRS. Therefore, we will prioritize variables that show associations with both mSOFA and mRS. This step will help us identify candidate features for clustering analyses by highlighting those most relevant to outcome variability. We will also apply hierarchical clustering to assess whether natural groupings exist in the data and perform principal component analysis to examine the dimensional structure and potential redundancies among features. These methods will provide insight into the underlying data structure and inform the number of potential patient subgroups before formal clustering.

In the final analytical phase, latent class analysis (LCA) and/or latent profile analysis (LPA) will identify patient subgroups based on the most informative early immunological and inflammatory variables selected during the exploratory phase. The association between LCA/LPA subgroups and mSOFA score will be assessed using linear mixed‐effects models, unadjusted first, then adjusted for baseline patient characteristics (e.g., age, sex, comorbidities) and Utstein‐style resuscitation variables. To better understand the longitudinal progression of organ dysfunction, we will cluster mSOFA trajectories over time into distinct groups. The resulting trajectory groups will be compared with LCA/LPA subgroups to determine whether specific immunological profiles are associated with different organ dysfunction patterns. Finally, we will assess the predictive value of LCA/LPA subgroup for 6‐month neurological outcome (mRS) using appropriate statistical models.

## 7 Planned Publications

1. Primary publication: clinical characteristics of the INNATUS cohort, proteomic data including cytokines, complement factors, and redox measurements.
2. Low density granulocytes and monocytes flow cytometry data, including their subpopulation frequencies and activation patterns.
3. Flow cytometry of cryopreserved circulating white blood cells with a focus on neutrophils, monocytes, and their activation signaling during early ICU care.
4. Spectral flow cytometry of PBMCs focusing on markers of immunosenescence during ICU treatment, including analysis of samples from the 6‐month recovery time point and comparison with a COVID‐19 cohort.
5. Comparison of flow cytometry of PBMCs and circulating inflammatory marker results between simultaneous samples obtained from arterial line and central venous catheter.
6. Liquid chromatography–mass spectrometry‐based proteomic analysis of enriched plasma proteins.
7. Blood total mRNA sequencing (focused on the first 24 h).

## 8 Discussion

Previous studies on inflammatory response after CA and ensuing whole‐body ischemia focus mostly on single inflammatory markers and their relation to patient outcomes. Thus, the inflammatory response in the context of CA, its progression over time, and its impact on organ failure development and long‐term outcomes remain poorly understood. In the INNATUS study, a systems immunology analysis of proteomic, transcriptomic, and flow cytometric data will be combined with comprehensive clinical data to gain a better understanding of the inflammatory response after CA. By measuring multiple inflammatory markers at consecutive time points, we aim to discover inflammatory trajectories that remain unnoticed when measuring single markers.

By choosing patients undergoing CABG surgery as a control group, we aim to distinguish the inflammatory response specific to CA. During CA, all organ systems are subjected to ischemia, whereas in CABG patients, transient ischemia affects predominantly cardiac tissues. In CA patients, thoracic trauma and pulmonary injury due to compressions and ventilation with 100% oxygen are also potential inducers of systemic inflammatory response. In CABG patients, surgical trauma and the use of heart‐lung machine are potential inducers of inflammation.

Post‐CA administration of methylprednisolone and tocilizumab, an IL‐6 receptor antagonist, resulted in decreased levels of IL‐6, CRP, and leukocyte counts, suggesting a modifiable target in post‐CA inflammation [6, 7]. A previous study has shown that therapeutic hypothermia results in lower IL‐6 levels [15]. However, in another study, the inflammatory responses did not differ between target temperatures of 33°C and 36°C [16]. To date, no anti‐inflammatory treatment has yet resulted in improved patient outcomes. Optimal targets for mean arterial pressure, body temperature management, and depth of sedation are being studied in a large multi‐center prospective study [17]. Combining these findings with the knowledge of the inflammatory characteristics of PCAS might provide future targets for anti‐inflammatory therapies in post‐CA care.

## Author Contributions

**Asser M. J. Seppä:** visualization, writing – original draft preparation. **Huai Hui Wong:** writing – review and editing. **Luz E. Cabrera:** writing – review and editing. **Dawit A. Yohannes:** methodology, writing – review and editing. **Maria Heliste:** writing – review and editing. **Marjaana Tiainen:** writing – review and editing. **Johanna Hästbacka:** writing – review and editing. **Markus B. Skrifvars:** writing – review and editing. **Eliisa Kekäläinen:** writing – review and editing. **Pirkka T. Pekkarinen:** conceptualization, methodology, project administration, supervision, writing – review and editing.

## Conflicts of Interest

The authors declare no conflicts of interest.

## Data Availability

Data sharing is not applicable to this article as no new data were created or analyzed in this study.
